# Proton Therapy for Partial Breast Irradiation: Rationale and Considerations

**DOI:** 10.3390/jpm11040289

**Published:** 2021-04-09

**Authors:** J. Isabelle Choi, Jana Fox, Richard Bakst, Shaakir Hasan, Robert H. Press, Arpit M. Chhabra, Brian Yeh, Charles B. Simone, Oren Cahlon

**Affiliations:** 1 Memorial Sloan Kettering Cancer Center, Department of Radiation Oncology, New York, NY 10065, USA; csimone@nyproton.com (C.B.S.II); cahlono@mskcc.org (O.C.); 2 New York Proton Center, New York, NY 10035, USA; jfox@montefiore.org (J.F.); richard.bakst@mountsinai.org (R.B.); shasan@nyproton.com (S.H.); rpress@nyproton.com (R.H.P.); achhabra@nyproton.com (A.M.C.); Byeh@nyproton.com (B.Y.); 3 Montefiore Medical Center, Department of Radiation Oncology, New York, NY 10467, USA; 4 Department of Radiation Oncology, Icahn School of Medicine at Mount Sinai, New York, NY 10029, USA

**Keywords:** breast cancer, proton therapy, precision medicine, partial breast irradiation, radiation therapy

## Abstract

In an era of continued advancements in personalized medicine for the treatment of breast cancer, select patients with early stage breast cancer may be uniquely poised to benefit from partial breast irradiation (PBI) delivered with proton therapy. PBI presents an opportunity to improve quality of life during treatment with a significantly shorter treatment duration. By targeting less non-target breast tissue, excess radiation exposure and resulting toxicities are also reduced. Proton therapy represents a precision radiotherapy technology that builds on these advantages by further limiting the normal tissue exposure to unnecessary radiation dose not only to uninvolved breast tissue but also the underlying thoracic organs including the heart and lungs. Herein, we present a concise review of the rationale for the use of proton therapy for PBI, evidence available to date, and practical considerations in the implementation and use of proton therapy for this indication.

## 1. Introduction

Breast cancer is the most common noncutaneous cancer, accounting for 30% of all female cancers [1]. There will be an estimated 281,550 new breast cancer diagnoses in the United States in 2021. Of these breast cancer cases, there has been an increasing proportion of women diagnosed at the early stages of presentation due to more widespread and consistent use of breast cancer screening over the past two decades [1,2]. Due to continued improvements in therapeutic strategies for early stage breast cancer (EBC), survival in this group of patients is excellent and in excess of 95–99% breast cancer-specific survival at 5 years [3,4]. Thus, the priority for these patients is to identify treatment methods that minimize toxicities and maximize quality of life, both during treatment and long-term.

Seminal clinical trials have demonstrated the equivalence of mastectomy and a breast-conserving treatment approach for patients with EBC, allowing for a less extensive surgical operation and retention of the patient’s native breast [5,6]. Thus, these patients are commonly treated with lumpectomy (also termed partial mastectomy, segmentectomy, or wide local excision) and lymph node sampling as indicated, followed by adjuvant radiation therapy (RT) to the involved breast. Systemic therapy using hormonal therapy and/or chemotherapy is also recommended based on specific patient and tumor characteristics.

Select patients with favorable EBC have the option to receive adjuvant RT using accelerated partial breast irradiation (PBI), an approach that presents advantages in its shorter and more convenient treatment course (ranging from a single day to 2 weeks, in comparison to 5–6 weeks for conventionally fractionated and3–4weeks for hypofractionated whole breast radiotherapy, WBRT) and in its ability to better spare uninvolved breast tissue from receiving full RT dose. Consensus guidelines have been developed by the American Society for Radiation Oncology (ASTRO) to help guide practitioners in identifying those patients most suitable for PBI [7]. Patient and tumor characteristics including tumor size, patient age, nodal status, disease stage, histology, grade, hormone receptor status, and surgical margins guide the recommended application of PBI, grouping patients into three categories: suitable, cautionary, and unsuitable. There are clinical trials ongoing to study the potential to identify expanded indications for PBI [8].

## 2. Review Methodology

For this review, we obtained data from the PubMed publicly available database (https://pubmed.gov, accessed on 23 March 2021). Following PRISMA guidelines for the conduct of a systematic review, we utilized broad search terms “breast”, “partial”, and “proton” to identify clinical series or trials on proton partial breast irradiation for inclusion. Of the 79 resulted publications, 40 were excluded as literature not involving proton therapy. Additional articles were excluded due to being review articles (n = 16), dosimetry/technique analyses (n = 10), case studies (n = 3), and not involving breast cancer (n = 1). A total of 9 studies related to proton partial breast irradiation were identified for inclusion in this review. The goal of this review is to assess published clinical literature to date on the novel application of proton therapy (PT) for the treatment of the partial breast to establish a foundation on which to build future investigations and gather additional data on this treatment approach.

## 3. Invasive Partial Breast Irradiation Techniques

PBI can be delivered using a variety of techniques. The more mature, invasive accelerated PBI techniques range from interstitial needle-based catheter brachytherapy to intracavitary balloon brachytherapy to intraoperative RT [9,10,11]. These applicators create a very conformal dose distribution immediately surrounding the surgical bed, thus targeting the area at highest risk of local relapse. However, given the invasive nature of this technique, there are also unique risks associated with these treatment deliveries compared with external beam RT, including hemorrhage, delayed wound healing, infection, pain, seroma formation, and the potential for suboptimal cosmesis [11]; additionally, disease control outcomes have been somewhat equivocal in large clinical trials to date. In the ELIOT trial, 1305 women were randomized to receive PBI with 21 Gy in 1 fraction of intraoperative RT vs WBRT with a conventional dose and fractionation (50 Gy plus a 10-Gy boost) [12]. At a median of 5.8 years follow-up, there was a 4.4% local recurrence rate in patients receiving IORT compared with 0.4% in the WBI arm, not meeting the definition of equivalence between the two treatment arms for this trial. In a similar investigation, the TARGIT-A trial randomized 1140 patients to receive IORT (20 Gy in 1 fraction) or WBI with standard fractionation typically of 40–56 Gy with or without a boost of 10–16 Gy. Although on initial reporting there were more local recurrences in the IORT arm, with longer-term follow-up, non-inferiority of IORT was confirmed for local control [13,14]. PBI with interstitial brachytherapy was studied in the GEC-ESTRO trial in which 1185 patients were randomized to WBI (50–50.4 Gy in 25–28 fractions) or interstitial brachytherapy (32 Gy in 8 fractions or 30.3 Gy in 7 fractions). At a median follow-up of 6.6 years, the rate of local recurrence was higher with PBI, although the rates were exceedingly low in both groups (1.44% PBI vs. 0.92% WBI, *p* = 0.42) [15].

## 4. External Beam Photon Partial Breast Irradiation

More recently, photon-based external beam PBI has been investigated as an attractive, non-invasive alternative approach to delivering PBI. However, here too, clinical results have not been consistent. Investigators from the University of Florence conducted a phase 3 trial randomizing 520 patients over the age of 40 years with EBC to receive WBRT to 50 Gy in 25 fractions or PBI to 30 Gy in 5 fractions [16]. There was no significant difference in the 5-year in-breast recurrence rate between arms, whereas acute and late toxicities, along with cosmesis, were superior in the PBI arm. In the NSABP B-39/RTOG 0413 phase 3 study, over 4200 patients were randomized to PBI (brachytherapy 34 Gy in 1 fraction (27%) or photon external beam 38.5 Gy in 3.85 Gy per fraction delivered twice daily over 5 days (73%)) vs conventional WBRT (50 Gy in 2 Gy per fraction delivered once daily over 5 weeks) [17]. Rates of disease control at 10 years were excellent in both groups (IBTR 4.6% PBI vs. 3.9% WBRT), but while the absolute difference was small, the PBI group did not meet the study criteria for equivalence to WBRT. The rate of grade 3 adverse events was also higher in the PBI group (10% PBI vs. 7% WBRT).

Both brachytherapy and photon-based external beam PBI possess distinct advantages, with brachytherapy providing superior conformality and normal breast tissue sparing, and photon PBI serving as a noninvasive treatment option that can avoid the perioperative pain and potential morbidities of brachytherapy. The increased tissue sparing with brachytherapy is a potentially significant advantage as a reduction in the amount of breast volume irradiated has been associated with a decrement in late cosmetic outcomes in photon PBI series [18].

## 5. Proton Partial Breast Irradiation

Proton therapy combines the advantages of both of these treatment approaches. Due to its unique physical properties and lack of exit dose, PT allows for a noninvasive PBI option that delivers a more conformal plan closer to that achieved with brachytherapy, not only benefitting normal breast tissue sparing and cosmesis, but also allowing for improved underlying heart and lung dose avoidance. This may be of particular benefit in subsets of patients with unfavorable anatomy, difficult tumor locations, or other characteristics that present a challenge in delivering an optimal photon or mixed photon/electron radiation plan. Patients with medially located tumors, especially those involving the left breast, often receive significant excess radiation dose to the heart with photon or photon/electron PBI due to requisite exit dose from the photon and electron beams needed to target the tumor bed with ample clinical margin. The long-term implications of this additional dose to the heart in the form of cardiac morbidity and excess major cardiac events, while numerically modest, can narrow the therapeutic index and limit the margin of benefit provided with radiotherapy for this select group of favorable EBC patients [19]. Patients with large resection cavities in a small breast may also be a particularly advantageous population in whom external beam photon RT may not be ideal. Although the partial breast is being targeted, with the limitation of beam angles available and exit dose with photon RT, significant portions of the breast may still be exposed to RT even while meeting standard PBI dose constraints, with potential negative consequences on cosmetic outcomes [18].

**Figure 1:**
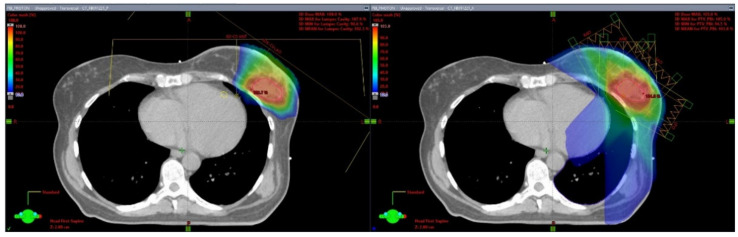
Proton and Photon Partial Breast Irradiation Treatment Plans. This is a patient with a left-sided breast cancer receiving partial breast irradiation. The left image is a representative axial slice of a proton plan using two treatment fields (anterior and left anterior oblique). The right image is a representative axial slice of a photon static-field intensity-modulated radiation therapy (IMRT) plan using 4 fields (right anterior oblique, anterior, left anterior oblique, and left posterior oblique).

Several institutional series have reported on the use of PT for PBI. Dosimetric studies have demonstrated the superiority of PT in sparing lungs, heart, and nontarget breast tissue compared with other external beam PBI modalities [20,21,22,23,24]. (Figure 1) PT also results in a far more homogeneous treatment plan compared with brachytherapy techniques, resulting in reduced maximum doses to the ipsilateral breast tissues, potentially reducing the risks of breast tissue fibrosis and fat necrosis [25].

Clinical experiences on the use of PT for PBI have been reported with increasingly promising results as proton technology has advanced and the optimal dose fractionation and treatment planning of PT PBI have been refined. (Table 1) In one of the earliest studies of PT PBI, investigators from Massachusetts General Hospital (MGH) treated patients with EBC with photon-based 3D PBI (n = 79) or double scattering PT (n = 19) [26,27]. Patients receiving PT were treated to 32 Gy (RBE) in 8 fractions delivered twice daily, with only 1 field treated per fraction. Compared with photon-based PBI, PT allowed for improved dosimetry, with significantly decreased heart (mean, maximum, D5%, D10%, and D20%), ipsilateral lung (mean, maximum, D5Gy, D5%, D10%, and D20%), and non-target breast dose. The rate of local failure at 7 years was not significantly different between the two groups (11% PT vs. 4% photon, *p* = 0.22), but there was significantly worse physician-reported late skin toxicity and adverse cosmesis noted with the use of PT [27,28].

A phase 2 trial from the National Cancer Center in Korea utilized passive scattering PT to treat 30 patients aged ≥40 years with tumors ≤3 cm and negative lymph nodes with PBI to 30 Gy (RBE) in 5 daily fractions [28]. The first 15 patients were treated using a single field, and the second 15 were treated with two fields in an attempt to decrease entrance dose to the skin. At 59 months of follow-up, there were no locoregional or distant recurrences, and all patients were alive. Overall, toxicities were limited. Physician cosmetic assessment was rated good or excellent in only 69% of patients at 3 years, although in patients treated with 2 fields, this increased to 89%. The suboptimal cosmesis for patients’ treatment with a single field seen in this series is consistent with that noted in the MGH experience, in which only one field was treated with each fraction. These findings suggest that caution should be taken when using with large fraction sizes, and that a single beam carrying a large dose to the skin and soft tissue of the breast may result in suboptimal cosmesis and undue toxicity for what should otherwise be a treatment with a favorable side effect profile.

Comparatively, in a phase 2 study from Loma Linda, 100 women with nonlobular carcinoma were also treated with passive scattering PT, but with a more moderately fractionated regimen of 40 Gy (RBE) in 10 daily fractions [30,31]. All patients were treated using 2–4 fields. At 5 years, local control was excellent at 97%. Toxicity outcomes were also favorable with no grade 3 or higher acute skin toxicities, and late toxicities limited to seven grade 1 events of telangiectasia. Patient and physician-reported cosmesis were good to excellent at 5 years in 90% without deterioration over time. In a cross-sectional study also from investigators at Loma Linda, quality of life (QOL) was evaluated using subjective instruments for 129 patients treated with PT PBI or photon whole breast irradiation (WBI). At 6.5 years post-diagnosis, measures of cosmesis, breast pain, breast texture, clothing fit, fatigue, and self-consciousness were significantly different between the two groups, in favor of the PT PBI cohort. [32]

A phase 2 multicenter study from the Proton Collaborative Group similarly treated 38 patients with PBI using 40 Gy (RBE) in 10 fractions using uniform scanning proton technology in the majority of patients. Locoregional control and overall survival were 100% at a median follow-up of 35 months, and there were no acute or late grade 3 or worse toxicities attributable to radiotherapy. Dose delivered to the heart and lungs were negligible, with the median volume of the heart receiving at least 5 Gy of 0% (V5Gy) and ipsilateral lung V20Gy and V10Gy of 0% and 0.17%, respectively [33].

Investigators at MD Anderson Cancer Center (MDACC) also conducted a phase 2 trial of 100 patients with EBC treated with passive scattering PT PBI to 34 Gy in 10 fractions delivered twice daily. At a planned interim analysis with a median follow-up of 24 months, local control and overall survival were both 100%, and there were no grade 3 or worse acute or late toxicities. The majority of patients and physicians rated cosmesis as good or excellent at 12 months (91% patients and 94% physicians). The most common late effect was telangiectasias (17%), and 7% of patients developed tumor bed retraction. Of note, heart and lung doses were exceedingly low, with a mean heart dose of 2 cGy (0.2–75.0 cGy) and mean ipsilateral lung dose of 19 cGy (range 0.2–164.0 cGy). [34]

In the only investigation of PT PBI exclusively using modern pencil beam scanning proton technology, the Mayo Clinic conducted a prospective study of 76 women with estrogen receptor-positive, node-negative EBC using a 3-fraction regimen of 7.3 Gy (RBE) per fraction using a two-field multi-field optimized approach. The skin dose was purposefully limited to a median maximum dose to 1 cc of the skin of 89% of the prescription dose; the heart and ipsilateral breast doses were also low at 0% and 28% of ipsilateral breast receiving ≥50% of the prescription dose, respectively. Early results have been reported, and at a median follow-up of 12 months, there have been no grade 2 or worse acute or late adverse events reported, and patient-reported cosmesis was good or excellent in 98% of patients [35].

These prospective investigations of the use of PT for PBI demonstrate that, with the appropriate dose regimen and treatment planning approach, PT can be an attractive treatment option for EBC. Tumor control with PT is excellent, and the toxicities associated with PT PBI are minimal. Cosmetic outcomes have improved with the increased recognition to utilize multi-field treatment plans and dose regimens that allow for ample time for repair between fractions [36]. While PT dosimetry is superior to other external beam PBI delivery methods, further improvements may be possible with increasing utilization of modern pencil beam scanning PT, which has increased ability to modulate dose to the skin, provide a homogeneous dose distribution, and more effectively minimize dose to nontarget tissue. Importantly, patient-reported quality of life also appears well preserved with PT PBI, with 90% of patients reporting a 7 or better QOL on a scale of 0–10, with 10 being the best QOL in the series from Mayo Clinic, and a patient satisfaction rate of treatment and results of 100% at 24 months in the study from MDACC [34,35].

## 6. Cost Effectiveness

While patients with left-sided tumors being treated to the internal mammary nodes are more susceptible to developing late cardiac toxicities from radiotherapy and thus may have significant clinical and cost effective benefit from PT [37], the cost effectiveness of PT for PBI has been called into question as a potential adverse factor in patient QOL and treatment accessibility. In the series from MD Anderson, the median out of pocket cost for patients was $700 (IQE $100–$1600), and the mean time away from work was 5 days (IQR2–5 days) for the 5-day treatment course. A cost effectiveness analysis of 8 schedules of PBI and WBRT revealed a lower total Medicare reimbursement for PT PBI ($13,833) compared with IMRT-WBI ($19,599) or multi-lumen brachytherapy PBI ($14,859), in contrast with the costs of the other 4 regimens evaluated ($6771–$13,149) [38]. These findings suggest minimal disruption in patient’s life from a financial standpoint, including considerations of cost and impact on livelihood.

## 7. Conclusions

With ongoing advancements in precision delivery of PT allowing for continued refinement of treatment approaches and further widening of the margin of benefit of proton PBI, this technique will likely continue to expand its role in the treatment of EBC. Further investigations will elucidate if a subset of patients may benefit most from the use of PT for PBI, such as those with large tumors, limited breast volume, and medial tumor location. In addition, patients with other special circumstances, such as unfavorable anatomy, connective tissue disorders, and prior irradiation, may also be considered more strongly for the use of PT for PBI.

## Figures and Tables

**Table 1:** Prospective investigations of partial breast irradiation (PBI) with proton therapy (PT).

Study	PT Type	N	Dose (Gy (RBE))	PT Details	MD-Reported Cosmesis	Patient-Reported Cosmesis	PT Toxicity	Disease Control
MGH [26,27]	Passive Scattering	19	32 Gy in 8 fx BID	1–3 fields (1 field treated per fx)	7 yr Good-excellent: 62%	7 yr Good-excellent 92%	69% Telangiectasias 54% Pigmentation changes 62% Other late skin tox	7 yr LF: 11% PP
NCC, Korea [29]	Passive Scattering	30	30 Gy in 5 fx QD	1–2 fields	3 yr Good-excellent: 69% (89% with 2-field plan)	--	Increased toxicity with single field plan	3 yr LF 0% 3 yr DF 0%
LLU [30,31]	Passive Scattering	90	40 Gy in 4 Gy × 10 fx QD	2–4 fields	5 yr Good-excellent: 90%	5 yr Good-excellent 90%	Acute: 62% Gr1–2RT dermatitis Late: 7% Gr 1 telangiectasia1% Fat necrosis	5 yr IBTR-FS 97% 5 yr OS 95%
PCG BRE007 [32,33]	Uniform Scanning Passive Scattering	37 1	40 Gy in 10 fx QD	≥3 fields (≥2 treated daily)	3 yr Good-excellent: 100%	BCTOS 4: 13.2% (nipple appearance, breast shape, scar tissue)	Gr 2–7 events ≥Gr 3–none	3 yr LF and DF: 0%
MDACC [34]	Passive Scattering	100	34 Gy in 10 fx BID	≥ 2 fields	2 yr Good-excellent: 84%	2 yr Good-excellent 96%	Acute Gr 2: 1% Breast pain 2% Hyperpigmentation 1% Pruritus 12% Dermatitis Late Gr 2: 5% Fatigue 1% Hyperpigmentation 2% Dermatitis	2 yr LF 0% 2 yr OS 100%
Mayo [35]	Pencil Beam Scanning	76	21.9 Gy in 3 fx QD	2 fields	NR	1 yr Good-excellent 98%	≥ Gr 2 acute or late tox-none	NR

Abbreviations: PT = proton therapy; PP = passive scattering proton therapy; RT = radiotherapy, fx = fractions; Gy = Gray; RBE = Radiobiological effective dose; yr = year; Gr = grade; LF = local failure; DF = distant failure; IBTR-FS ipsilateral breast tumor recurrence-free survival; OS = overall survival; NR = not yet reported; QD = daily; BID = twice daily.

## Data Availability

No new data were created or analyzed in this study. Data sharing is not applicable to this article.
